# A Hypoxia Signature for Predicting Prognosis and Tumor Immune Microenvironment in Adrenocortical Carcinoma

**DOI:** 10.1155/2021/2298973

**Published:** 2021-09-21

**Authors:** Xi Chen, Lijun Yan, Yu Lu, Feng Jiang, Ni Zeng, Shufang Yang, Xianghua Ma

**Affiliations:** ^1^Department of Endocrinology, Taizhou Clinical Medical School of Nanjing Medical University (Taizhou People's Hospital), Taizhou, Jiangsu, China; ^2^Department of Hepatology, Nantong Third People's Hospital Affiliated to Nantong University, Nantong, Jiangsu, China; ^3^Department of Neonatology, Obstetrics and Gynecology Hospital of Fudan University, Shanghai, China; ^4^Department of Dermatology, Affiliated Hospital of Zunyi Medical University, Zunyi, Guizhou, China; ^5^Department of Nutriology, The First Affiliated Hospital of Nanjing Medical University, Nanjing, Jiangsu, China

## Abstract

Adrenocortical carcinoma (ACC) is a rare malignancy with dismal prognosis. Hypoxia is one of characteristics of cancer leading to tumor progression. For ACC, however, no reliable prognostic signature on the basis of hypoxia genes has been built. Our study aimed to develop a hypoxia-associated gene signature in ACC. Data of ACC patients were obtained from TCGA and GEO databases. The genes included in hypoxia risk signature were identified using the Cox regression analysis as well as LASSO regression analysis. GSEA was applied to discover the enriched gene sets. To detect a possible connection between the gene signature and immune cells, the CIBERSORT technique was applied. In ACC, the hypoxia signature including three genes (CCNA2, COL5A1, and EFNA3) was built to predict prognosis and reflect the immune microenvironment. Patients with high-risk scores tended to have a poor prognosis. According to the multivariate regression analysis, the hypoxia signature could be served as an independent indicator in ACC patients. GSEA demonstrated that gene sets linked to cancer proliferation and cell cycle were differentially enriched in high-risk classes. Additionally, we found that PDL1 and CTLA4 expression were significantly lower in the high-risk group than in the low-risk group, and resting NK cells displayed a significant increase in the high-risk group. In summary, the hypoxia risk signature created in our study might predict prognosis and evaluate the tumor immune microenvironment for ACC.

## 1. Introduction

Adrenocortical carcinoma (ACC) is a rare malignant endocrine tumor arising from the cortex of the adrenal gland, which has a dismal prognosis [1]. The Surveillance, Epidemiology, and End Results (SEER) registry reported that the incidence of ACC in the United States was one per million people annually from 1974 to 2014 [2]. Although radical surgical resection is the most effective therapy, 5-year survival rates of ACC patients range from 15% to 44% [3]. Therefore, it is warranted to identify an improved prognostic feature to predict the prognosis for ACC patients and then assign them to appropriate therapeutic interventions.

Hypoxia arising from decreased oxygen supply is one of the hallmarks of tumor microenvironment. Tumor hypoxic condition is closely correlated with proliferation, tumor recurrence, metastasis, drug resistance, and decreased patient survival [4]. There are various hypoxia-associated genes with prognostic power in cancer, such as P4HA1 in glioblastoma [5] and PDSS1 in hepatocellular carcinoma [6]. Currently, the impact of tumor microenvironment on the immune system has paid great attention. It is well known that hypoxia is regarded as an immune suppressor on immune system. The novel hypoxia risk signature developed by Lin et al. was thought to be an independent prognostic indicator and a tool for measuring immune microenvironment for glioma patients [7]. Another hypoxia-related model established by Shou et al. was a predictor for the immune microenvironment in Melanoma [8]. Hence, hypoxia genes could be considered as latent biomarkers for evaluating immune microenvironment in cancers.

Over the last decades, advances in epigenetic analyses and genome-wide expression profile studies had provided us with a better understanding of the molecular genetics of ACC. Several biomarkers associated with metastasis, prognosis, and survival in ACC patients have been confirmed by data mining. However, hypoxia signature for predicting ACC prognosis has not been established. Therefore, we aimed to identify a potential hypoxia risk signature based on the hypoxia-associated genes, which could be considered as a robust prognostic tool to evaluate the immune microenvironment for ACC patients. In years to come, the risk model might be applied to help physicians quickly identify prognosis and make important treatment decisions in ACC.

## 2. Materials and Methods

### 2.1. Datasets

The RNA-seq data and relevant clinical information of ACC patients were downloaded from the Cancer Genome Atlas (TCGA) and Gene Expression Omnibus (GEO) database (GSE19750). RNA-seq data for normal adrenal tissue were obtained from the GTEx database. More clinical data details were provided in Supplementary Material Table S1 as in our previous published study [9]. We searched the Molecular Signatures Database for getting the collection of hypoxia-related genes. No ethical approval was required because the data we utilized were obtained from public databases.

### 2.2. Construction of Hypoxia Signature

The univariate Cox regression analysis was performed to evaluate the relationship of hypoxia genes with overall survival (OS) in ACC. Then, we further applied the glmnet package in *R* to perform the LASSO regression analysis for narrowing the range of genes whose *P* value <0.05 in the univariate analysis. Multivariable Cox regression was then utilized to gain the coefficients. Formula for calculating the risk score is as follows:

(1) $$\begin{array}{c} \text{Risk score}=\sum_{i=1}^{n}\left(\text{coef mRNAi}*\text{expression of mRNAi}\right), \end{array}$$

where coef is the coefficient calculated by multivariable Cox regression.

### 2.3. Survival Analysis and Constitution of a Predictive Nomogram

We applied the Kaplan–Meier analysis to make a comparison between the two groups in TCGA and GEO cohorts. To find the possible prognostic variables, the univariate Cox analysis was done. Additionally, the multivariate Cox analysis was utilized to identify whether the risk signature could be considered as an independent risk factor for OS in ACC. The precision of the risk model in forecasting the survival of ACC was validated using a ROC curve. A predictive nomogram was built based on *T* and risk score to estimate the prognosis at 1-, 3-, and 5-year for ACC.

### 2.4. Gene Set Enrichment Analysis (GSEA)

GSEA was used to find a substantial variation in gene sets presented in the two risk groups. Under each analysis, 1000 times of gene set permutations were performed. A risk score was calculated using the phenotype label. Significant gene sets were classified as those with normalized enrichment score >1 and minimal *P* value <0.05.

### 2.5. Estimation of Immune Cell Subtype Proportion

Newman et al. developed an analytical tool named CIBERSORT, which can offer a way for estimating the content of immune cells through the expression of each gene [10]. To further estimate the proportions of 22 human immune cells in the two risk groups, we normalized the mRNA expression matrix and utilized the analytical tool CIBERSORT in ACC cohorts.

## 3. Results

### 3.1. Establishment of Hypoxia Risk Signature

**Figure 1:**
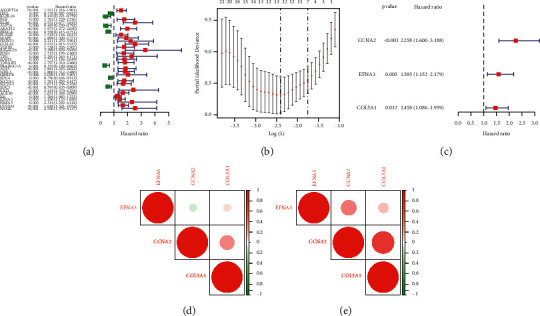
Establishment of hypoxia risk signature in ACC patients. (a) Prognostic genes identified by univariate Cox regression. (b) LASSO regression algorithm. (c) The hypoxia risk signature developed by multivariate Cox regression. (d)-(e) Comparison of Spearman's correlation coefficient among 3 hypoxia-associated genes.

To establish a hypoxia risk signature and explore its prognostic value in ACC patients, a total of 144 overlapping hypoxia-associated genes derived from the two cohorts were selected for the following analysis. As illustrated in Figure 1(a), a total of 33 hypoxia-associated genes strongly correlated with the OS rate were identified by the univariate Cox analysis. Finally, 13 hypoxia-associated genes were retained via the LASSO regression analysis (Figure 1(b)). In addition, a prognostic model was established by the multivariate Cox regression analysis (Figure 1(c)), which was composed of 3 genes: CCNA2, EFNA3, and COL5A1. The formula for risk score calculation is as follows: risk score=(0.81*∗*CCNA2)+(0.46*∗*EFNA3)+(0.38*∗*COL5A1). Thereafter, total patients were allocated, respectively, into the low- and high-risk groups in two cohorts according to their risk score values. It was found that all 3 genes were correlated with one another in both TCGA and GEO cohorts (Figures 1(d) and 1(e)). The process was shown in the Supplementary Material Figure S1. As shown in Figure S2, we further compared the expressional levels of 3 genes in ACC tissues with that in 127 normal adrenal tissues from the GTEx database and found that all 3 genes were differentially expressed in ACC tissues and normal tissues (*P* < 0.001).

### 3.2. Effect of the Hypoxia-Related Signature on the Prognosis of ACC Patients

**Figure 2:**
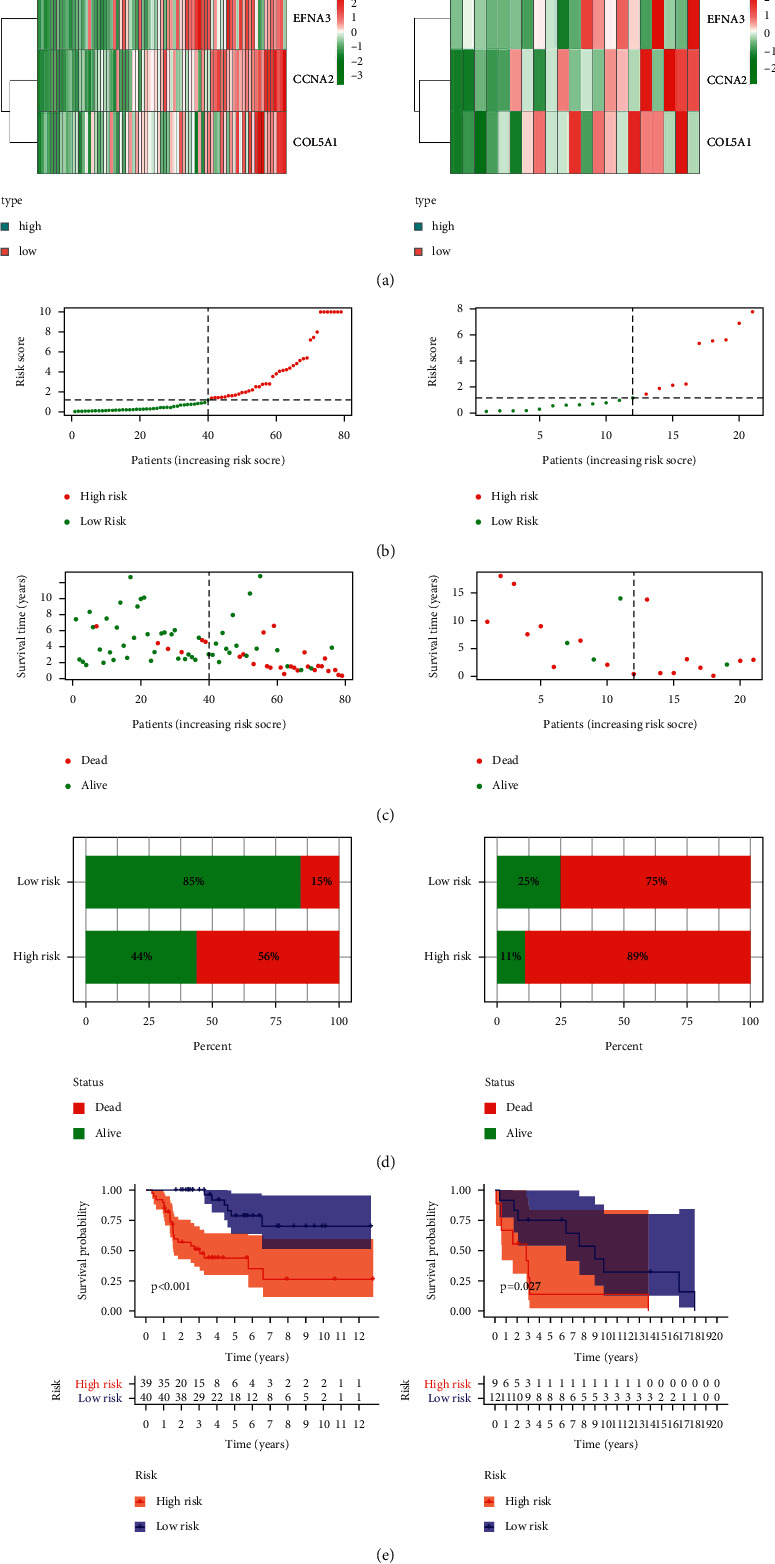
Application value of the hypoxia-related signature in predicting the outcomes of ACC patients. (a) Heatmap of expressional profiles of 3 hypoxia-associated genes in the high/low-risk group in two cohorts. (b)-(c) the risk score and OS in patients in the high/low-risk group in two cohorts. (d) The death rate in the high/low-risk group. (e) Kaplan–Meier survival analysis of patients in the high/low-risk group.

The expressional levels of 3 hypoxia-associated genes were correlated with a higher risk score in TCGA and GEO cohorts, as seen in the heatmap, suggesting that the patients with a higher risk score were more likely to have an anoxic microenvironment (Figures 2(a) and 2(b)). As illustrated in Figures 2(c) and 2(d), there was a significantly higher death rate in the high-risk group compared with that in the low-risk group. Furthermore, the effect of the hypoxia-related signature on the prognosis of ACC patients was assessed by the Kaplan–Meier (KM) analysis. It was also found that the OS of patients in the high-risk group was obviously lower than that of patients in the low-risk group in TCGA cohort (*P* < 0.001), which was further validated in the GEO cohort (*P*=0.027). These results supported the hypothesis that the new hypoxia-related signature had a definite effect in predicting the outcomes of ACC patients.

### 3.3. Correlation between Hypoxia-Associated Gene and Clinicopathological Features in ACC Patients

**Figure 3:**
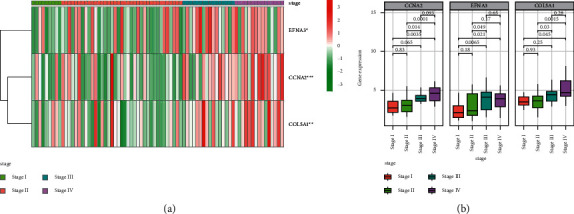
Correlation between hypoxia-associated gene and stages of ACC. (a) Heatmaps of expressional profiles of 3 hypoxia-associated genes at different stages from TCGA cohort. (b) The expressional levels of hypoxia-associated genes in ACC patients at different stages.

Taking into account the significant biological roles of hypoxia in the occurrence and progression of cancers, the correlation of 3 hypoxia-associated genes included the risk signature with the pathological stages in ACC patients. As shown in Figure 3, the expressional levels of 3 hypoxia-associated genes were obviously higher in ACC patients at the advanced stage.

### 3.4. Obvious Effect of Hypoxia Risk Signature in Predicting the Outcomes of ACC Patients

**Figure 4:**
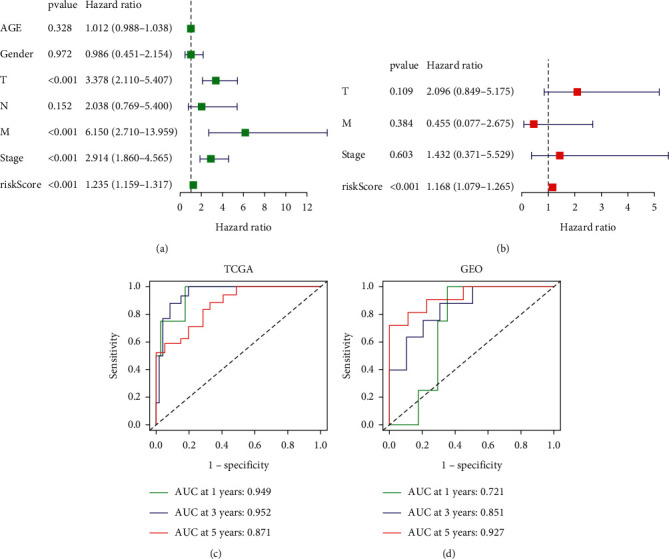
Effect of the hypoxia risk signature in predicting the outcomes of ACC patients. (a)-(b) Evaluation of the independent prognostic effect of the hypoxia-related signature in TCGA cohort by univariate and multivariate Cox regression analyses. (c)-(d) ROC curves for assessing the effect of the hypoxia risk signature in predicting the outcomes of ACC patients in two cohorts.

The univariate regression analysis indicated that a higher risk score was correlated with a poorer OS (*P* < 0.001). *T*, *M*, and stage were other factors linked to poor OS rate (Figure 4(a)). The multivariate analysis showed that a higher hypoxia risk score was independently correlated with a poorer OS rate (*P* < 0.001), suggesting that it may be considered to be one of independent prognostic factors for ACC patients (Figure 4(b)). Based on the data from TCGA and GEO cohorts, the received operating characteristic (ROC) curve was drawn to evaluate the predictive effect of the hypoxia risk signature. The area under the curve (AUC) of 1-, 3-, and 5-year OS rates in TCGA cohort were 0.949, 0.952, and 0.871, respectively, suggesting that the risk signature had an obvious effect in predicting the outcomes of ACC patients (Figure 4(c)). It had been furthermore confirmed in GEO cohort (Figure 4(d)).

### 3.5. Development of a Predictive Nomogram

**Figure 5:**
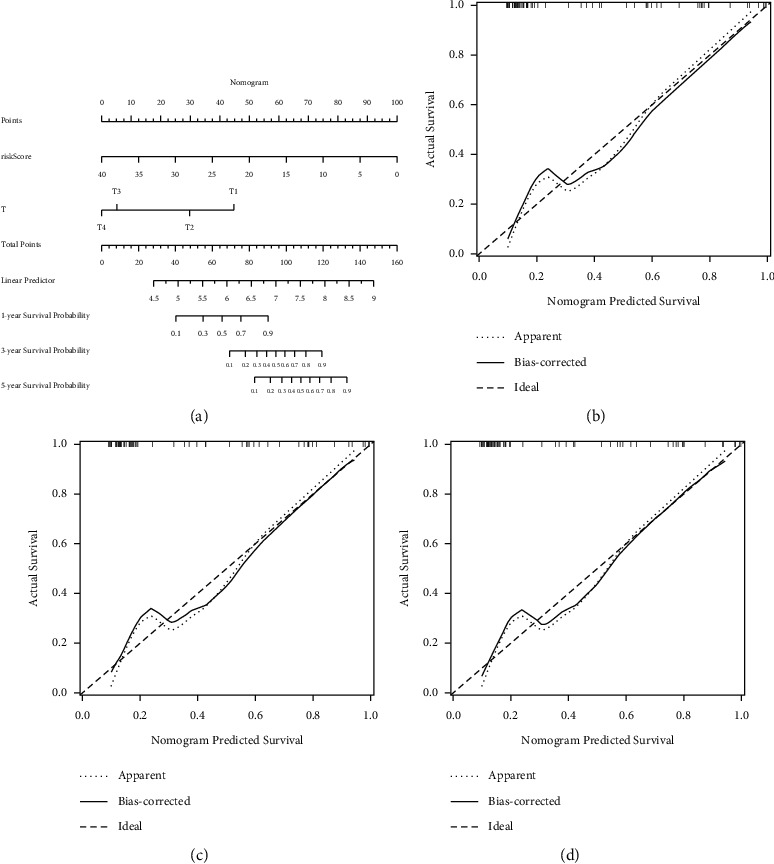
Development of a predictive nomogram for ACC patients in TCGA cohort. (a) Nomogram for predicting prognosis of ACC patients in TCGA cohort. (b)–(d) Calibration plots for predicting probabilities of the nomogram at the 1, 3, and 5 years.

In order to develop a convenient tool to predict the outcomes of ACC patients in clinical practice, a predictive nomogram based on *T* and a risk score based on TCGA cohort were constructed (Figure 5(a)). As illustrated in calibration plots, as an optimal model, the nomogram created in this study had reasonable precision (Figures 5(b)–5(d)).

### 3.6. Hypoxia-Associated Signaling Pathways Screened by GSEA

**Figure 6:**
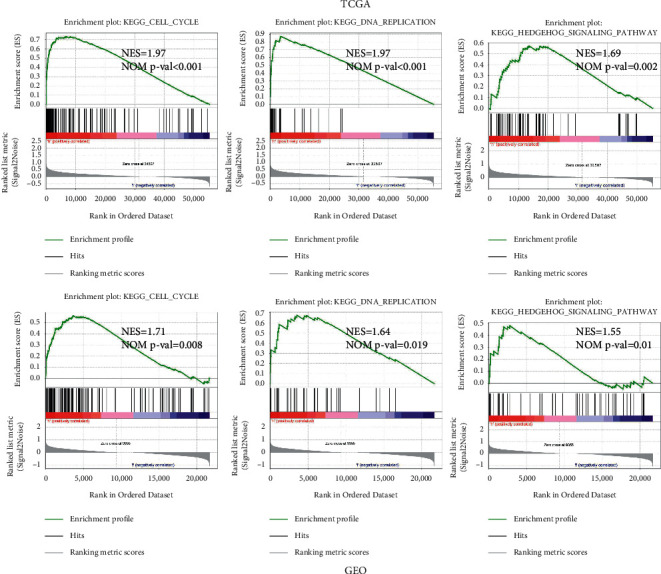
Hypoxia-associated signaling pathways screened by GSEA. (a) Gene sets enriched in the high-risk group performed by GSEA in TCGA cohort. (b) Gene sets enriched in the high-risk group performed by GSEA in GEO cohort.

The hypoxia-related signaling pathways were compared between the high- and low-risk groups by the GSEA analysis. Gene sets linked to cancer proliferation and cell cycle, such as cell cycle, DNA replication, and Hedgehog signaling, were highly enriched in the high-risk group in TCGA cohort (Figure 6(a)). These findings were further validated in the high-risk group in the GEO cohort (Figure 6(b)).

### 3.7. Correlation of Hypoxia Risk Signature with Immunity Microenvironment

**Figure 7:**
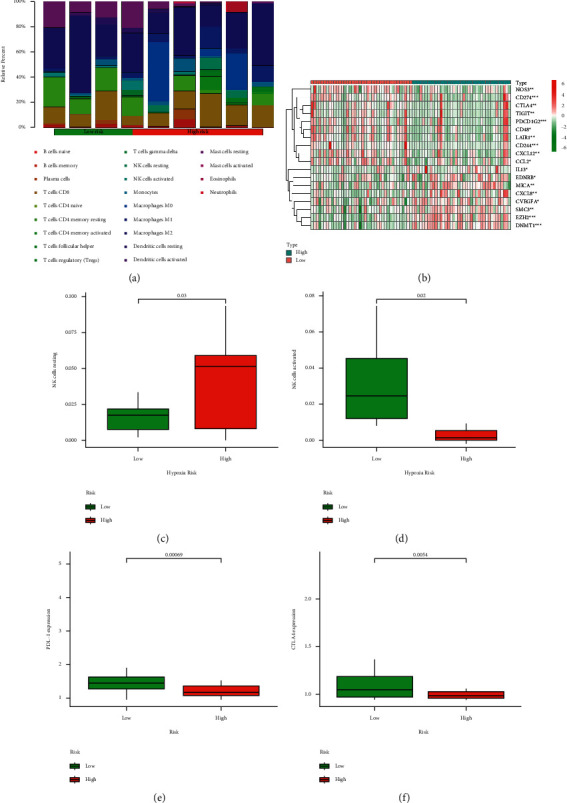
Correlation of hypoxia risk signature with immunity microenvironment. (a) Presence of immune cellular infiltration in the high/low-risk group. (b) Heatmap of the genes for negative regulation of the cancer-immunity cycle in the high/low-risk group in TCGA cohort. (c)-(d) Percentages of resting and activated NK cells in the high/low-risk group. (e)-(f) PDL1 and CTLA4 expressional levels in the high/low-risk group.

It is suggested that the tumors in a hypoxic microenvironment could be exempted from physical antitumor immune responses due to inhibited antineoplastic immune cells and promoted tumor immune escape. In our study, the potential of a hypoxia risk signature was investigated to determine the immunity microenvironment; variations in immune infiltration of 22 immune cell types were evaluated and compared between ACC patients in the low- and high-risk groups using the CIBERSORT tool and LM22 signature matrix. The findings from TCGA cohort are presented in Figures 7(a)–7(d). The expressional levels of the genes for negative regulation of the cancer-immunity cycle were detected in the low/high-risk groups by querying gene expression signatures collected from Tracking Tumor Immunophenotype website [11]. The expression levels of the genes for negative regulation of the cancer-immunity cycle were upregulated in the high-risk group, suggesting that the cancer-immunity cycle in patients of the high-risk group was not activated. In addition, the percentage of resting NK cells was obviously greater in the high-risk group, while the content of activated NK cells was decreased. Previous studies showed that the expression levels of immune checkpoints were related to hypoxia. Thus, the expression levels of immune checkpoints in the low/high-risk groups were further investigated in this study. It was found that the expressional levels of critical immune checkpoints such as programmed death ligand-1 (PDL1) and cytotoxic T lymphocyte antigen-4 (CTLA4) were obviously lower in the high-risk group than in the low-risk group (Figures 7(e) and 7(f)).

## 4. Discussion

ACC is a rare malignancy originating from the adrenal cortex with a dismal prognosis. In recent years, accumulating studies have confirmed that several biomarkers are prognostic factors of ACC. It is reported that tumors with the expression of Ki67 higher than 10% have significantly poor prognosis than those with lower than 10% in ACC patients [12]. The nuclear division cycle 80, cyclin B2, and miRNAs have been reported to involve in carcinogenesis and progression of ACC, predicting OS in patients with ACC [13, 14]. Additionally, PTTG1 and GLUT1 had been proven as a marker of poor survival in ACC [15, 16]. However, a single gene biomarker could be affected by various factors leading to an incorrect predictive effect, and some studies have found that gene signatures could offer a better alternative for predicting prognosis and survival [17]. Thus, it is necessary to find more efficient and sensitive gene signature comprising various genes to predict ACC patients' outcomes.

There are several reported risk models based on multiple genes having the prognostic value in ACC patients with bioinformatics methods. For example, the study of the weighted gene coexpression network analysis and algorithm analysis constructed a gene coexpression network associated with tumor grade and poor prognosis in ACC. Results have accentuated 12 hub genes with good distinctive power for malignancy and correlated with unfavorable prognosis and tumor stages [18]. Fu et al. reported that the immune risk signature based on 30 immune-associated genes linked to OS could predict prognosis for patients with ACC [19]. In our research, by using the bioinformatics analysis, we first built a novel hypoxia-related signature including only three genes and demonstrated that the hypoxia risk signature had a powerful value in predicting ACC patients' survival. Compared with the above models consisting of many genes, it is convenient for physicians to apply our risk model in clinic, which contains only three hypoxia-related genes. Moreover, the hypoxia genes involved in the risk signature were identified through univariate and multivariate Cox regression analyses together with the LASSO regression analysis, suggesting that genes participated in the signature had explicit power compared with other known biomarkers in predicting prognosis for ACC.

According to previous studies, the three genes (cyclin A2, COL5A1, and EFNA3) included in our risk signature were involved in tumorigenesis and hypoxia microenvironment. The expression of ephrin-A3 (EFNA3), a member of the ephrin family, significantly increased under the ischemic-hypoxic condition [20]. EFNA3 has been reported to promote the metastatic ability in breast cancer [21]. Cyclin A2 (CCNA2) belongs to a strongly conserved cyclin family, promoting cell cycle transition in cancers. There is accumulating evidence demonstrating the correlation between CCNA2 and tumorigenesis of numerous cancers, including lung cancer, breast cancer, and pancreatic ductal carcinoma [22–24]. Moreover, CCNA2 has been implicated in the metastasis, recurrence, and poor prognosis of ACC [25]. Collagen, type V, alpha 1 (COL5A1), one of the collagen family, can promote tumor growth as an oncogenic protein in cancers [26]. As hypoxia is known to be linked with more violent cancer phenotypes, we further examined the predictive performance of the hypoxia signature for ACC patients' OS. In our study, the hypoxia risk signature established by 3 hypoxia-associated genes was an independent factor in predicting OS for ACC, further supporting the idea that the hypoxia risk signature can offer a more targeted and powerful prediction than a single biomarker. Additionally, nomogram constructed in our study could be a more classification tool for allowing clinicians to make more accurate predictions of ACC survival.

Hypoxia is one of the characteristics of malignant tumor, which results from the imbalanced oxygen supply. Apart from promoting malignant tumors development and progression, hypoxia also takes part in antitumor immune effects through reducing proliferation of lymphocytes, including T cells, B cells, and natural killer (NK) cells [27]. Consistent with the previous study, our findings revealed that the content of resting NK cells increased in the high hypoxia risk group, and the proportion of activated NK cells declined, presenting an immune suppressive condition in the high-risk group of ACC patients. Immunotherapy has paid more and more attention for which can present an antitumor role in the process of cancer treatment. Whereas, according to recent studies, tumor cells can avoid the immune response by utilizing various immune checkpoints, which can play a crucial part in cancer immunotherapy, including programmed death-1 (PD1), PDL1, and CTLA4. A previous study demonstrated that PDL1 is expressed in the cytomembrane of cancer cells. Meanwhile, high PDL1 mRNA expression was correlated with longer disease-free survival (DFS) [28]. Consistent with the above findings, the lower expression of immune checkpoint PDL1 and CTLA4 in the high-risk group was linked with the poor prognosis in our study.

As far as we know, this is the first study aiming at developing and validating a hypoxia risk signature in ACC. Our results revealed that the signature could be utilized as a promising tool for predicting prognosis and reflecting the immune microenvironment in ACC. Different from previous studies, this model focused on hypoxia-associated genes. However, some limitations in our study should be noted. Owing to the limited number of ACC patients, we could not conduct a further stratified analysis, which is one limitation of our study. More prospective research studies with larger sample are needed for further validation of the prognostic performance. Besides, the definite function of the hypoxia signature was not verified by functional experiments. Thus, several further steps must be completed before these findings can be extended to clinical practice.

## Data Availability

RNA-seq data and clinical information applied to support the findings of this study were downloaded from the Cancer Genome Atlas (TCGA) (https://cancergenome.nih.gov/), Gene Expression Omnibus (GEO) repository (GSE19750), and GTEx (https://www.gtexportal.org/).

## Supplementary Materials

Supplementary Materials Figure S1: flowchart of the study. Table S1: clinical characteristics of ACC patients in TCGA and GEO. Figure S2: the expression of CCNA2, EFNA3, and COL5A1 in ACC tissues and normal adrenal tissues.
