# Supplementary figures and images for: A Hypoxia Signature for Predicting Prognosis and Tumor Immune Microenvironment in Adrenocortical Carcinoma
Source: J Oncol. 2021 Sep 21;2021:2298973. doi: 10.1155/2021/2298973 (PMC8481041; doi:10.1155/2021/2298973)

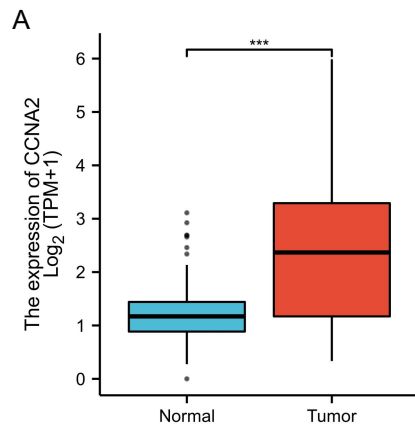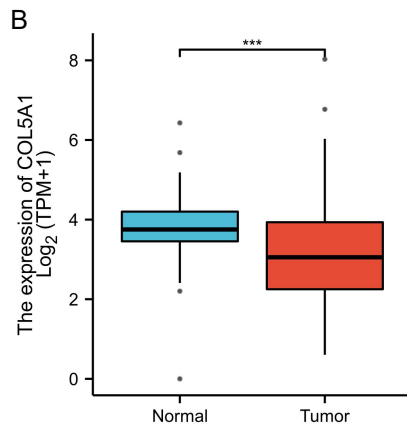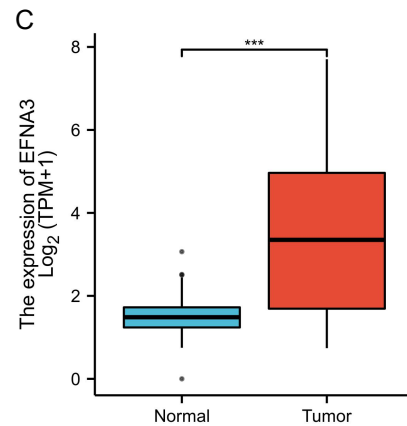

Supplement: Supplementary Materials — Figure S1: flowchart of the study. Table S1: clinical characteristics of ACC patients in TCGA and GEO. Figure S2: the expression of CCNA2, EFNA3, and COL5A1 in ACC tissues and normal adrenal tissues. [file 2298973.f1.zip › 2298973.f1/Supplementary Figure S2.pdf]
