# Supplementary material for: A Hypoxia Signature for Predicting Prognosis and Tumor Immune Microenvironment in Adrenocortical Carcinoma
Source: J Oncol. 2021 Sep 21;2021:2298973. doi: 10.1155/2021/2298973 (PMC8481041; doi:10.1155/2021/2298973)
Supplement: Supplementary Materials — Figure S1: flowchart of the study. Table S1: clinical characteristics of ACC patients in TCGA and GEO. Figure S2: the expression of CCNA2, EFNA3, and COL5A1 in ACC tissues and normal adrenal tissues. [file 2298973.f1.zip › 2298973.f1/Supplementary Table S1.doc]

**Table S1 Clinical Characteristics of ACC patients in the TCGA and GEO cohorts**

| Characteristics | Subgroup | TCGA(n=79) | GEO(n=21) |
| --- | --- | --- | --- |
| Age | ≥50 | 39 | 14 |
|  | ＜50 | 40 | 7 |
| Gender | Female | 49 | 10 |
|  | Male | 30 | 11 |
| Survival status | Dead | 29 | 17 |
|  | Living | 50 | 4 |
| Stage | Stage1 | 9 | 1 |
|  | Stage2 | 36 | 7 |
|  | Stage3 | 16 | 1 |
|  | Stage4 | 16 | 4 |
|  | Recurrence | - | 8 |
|  | unknown | 2 | - |
| T | T1 | 9 | - |
|  | T2 | 41 | - |
|  | T3 | 8 | - |
|  | T4 | 19 | - |
|  | unknown | 2 | - |
| N | NO | 68 | - |
|  | N1 | 9 | - |
|  | unknown | 2 | - |
| M | M0 | 61 | - |
|  | M1 | 16 | - |
|  | unknown | 2 | - |
